# Evidence-based clinical practice guidelines for prevention, screening and treatment of peripartum depression

**DOI:** 10.1192/bjp.2025.43

**Published:** 2025-11

**Authors:** Sandra Nakić Radoš, Ana Ganho-Ávila, Maria F. Rodriguez-Muñoz, Rena Bina, Sarah Kittel-Schneider, Mijke P. Lambregtse-van den Berg, Ilaria Lega, Angela Lupattelli, Greg Sheaf, Alkistis Skalkidou, Ana Uka, Susanne Uusitalo, Laurence Bosteels-Vanden Abeele, Mariana Moura-Ramos

**Affiliations:** Department of Psychology, Catholic University of Croatia, Zagreb, Croatia; Centre for Research in Neuropsychology and Cognitive Behavioural Interventions, Faculty of Psychology and Educational Sciences, University of Coimbra, Coimbra, Portugal; Department of Psychology, Universidad Nacional de Educación a Distancia (UNED), Madrid, Spain; School of Social Work, Bar Ilan University, Ramat Gan, Israel; Department of Psychiatry and Neurobehavioural Science, School of Medicine, University College Cork, Cork, Ireland; APC Microbiome, University College Cork, Cork, Ireland; Department of Psychiatry, Psychosomatic Medicine and Psychotherapy, University Hospital of Würzburg, Würzburg, Germany; Departments of Psychiatry and Child & Adolescent Psychiatry, Erasmus University Medical Center, Rotterdam, The Netherlands; National Centre for Disease Prevention and Health Promotion, Istituto Superiore di Sanità, Rome, Italy; Department of Pharmacy, University of Oslo, Oslo, Norway; The Library of Trinity College Dublin, Dublin, Ireland; Department of Women’s and Children’s Health, Uppsala University, Uppsala, Sweden; Outpatient Gynaecological Department, Akademiska University Hospital, Uppsala, Sweden; Department of Nursing and Physiotherapy, Western Balkans University, Tirana, Albania; Department of History, Culture and Communication Studies / Philosophy, University of Oulu, Oulu, Finland; Make Mothers Matter, EU Delegation, Brussels, Belgium; Clinical Psychology Unit, Hospitais da Universidade de Coimbra, Unidade Local de Saúde de Coimbra, Coimbra, Portugal

**Keywords:** Perinatal depression, screening, prevention, treatment, clinical guidelines

## Abstract

**Background:**

Peripartum depression (PPD) is a prevalent mental health disorder in the peripartum period. However, a recent systematic review of clinical guidelines relating to PPD has revealed a significant inconsistency in recommendations.

**Aims:**

This study aimed to collect up-to-date evidence on the effectiveness of interventions and provide recommendations for prevention, screening and treating PPD.

**Method:**

A series of umbrella reviews on the effectiveness of PPD prevention, screening and treatment interventions was conducted. A search was performed in five databases from 2010 until 2023. The guidelines were developed according to the GRADE framework and AGREE II Checklist recommendations. Public stakeholder review was included.

**Results:**

One hundred and forty-five systematic reviews were included in the final analysis and used to form the guidelines. Forty-four recommendations were developed, including recommendations for prevention, screening and treatment. Psychological and psychosocial interventions are strongly recommended for preventing PPD in women with no symptoms and women at risk. Screening programmes for depression are strongly recommended during pregnancy and postpartum. Cognitive–behavioural therapy is strongly recommended for PPD treatment for mild to severe depression. Antidepressant medication is strongly recommended for treating severe depression in pregnancy. Electroconvulsive therapy is strongly recommended for therapy-resistant and life-threatening severe depression during pregnancy. Other recommendations are offered to healthcare professionals, stakeholders and researchers in managing PPD in different contexts.

**Conclusion:**

Treatment recommendations should be implemented after carefully considering clinical severity, previous history, risk–benefit for mother and foetus/infant and women’s values and preferences. Implementation of evidence-based clinical practice guidelines within country-specific contexts should be facilitated.

Peripartum depression (PPD) is formally defined as an episode of unipolar major depressive disorder (MDD) with onset during pregnancy or within 4 weeks after childbirth.^1^ However, a recent review pointed out several specificities of PPD compared with MDD, such as distinctive risk factors (e.g. ovarian tissue expression, premenstrual syndrome), symptomatology (less sad mood, lower suicidal risk, but predominant anxiety, irritability, obsessive thoughts and guilt about not being a good mother) and wider cross-cultural variety. Furthermore, it showed that PPD can occur up to 1 year postpartum.^2^ Therefore, for the purpose of this document, a broad definition of PPD was adopted, including the pregnancy and the postpartum period up to 1 year after childbirth. We use the terms ‘depression during pregnancy’ when we refer to prepartum depression only and ‘postpartum depression’ when referring only to depression after childbirth.

## PPD global prevalence and its impact

The prevalence of PPD varies across countries and regions, and there are several factors that influence its occurrence. According to comprehensive meta-analyses, the overall global prevalence rate of depression during pregnancy is 20%^3^ and that of postpartum depression is 17.4%. PPD^4^ has many adverse effects, and a systematic review of 122 studies documented the negative impact of PPD on mother, infant and the mother–infant relationship.^5^ Maternal depression has, completely justifiably, been called a ‘thief that steals motherhood’.^6^

In addition to the consequences of PPD for the mother, the child/children and their family, it is important to note that PPD has significant economic costs to society. In an economic report about the lifetime costs of PPD and anxiety,^7^ the estimated total cost is more than £75 000 (about €88 000 in 2024) per woman diagnosed with PPD.

## Limitations of existing guidelines

To prevent PPD and offer timely screening followed by appropriate treatment, it is crucial to have clinical practice guidelines to provide instruction on all these steps, from prevention to screening, and treatment and addressing the different evidence-based options available. A recent systematic review of clinical guidelines relating to PPD, which includes guidelines such as those provided by the National Institute for Health and Care Excellence (NICE),^8^ has revealed a significant inconsistency in recommendations.^9^ Furthermore, most analysed guidelines demonstrated a high level of bias. Also, information on prevention was scarce. Regarding treatment, most clinical guidelines were focused on pharmacological treatment, less on psychological treatment, and even more rarely on other treatment options,^9^ such as non-invasive brain stimulation and alternative and complementary therapies. Also, none of the existing guidelines, including the most recent ones by the American College of Obstetricians and Gynaecologists,^10,11^ specifically focus on PPD.

## Aims

Considering that PPD is a highly prevalent mental health disorder in the peripartum period, and given a large amount of new evidence in this field and a lack of consistency in the previous guidelines with a high risk of bias,^9^ the aim of this paper was to collect and summarise the current evidence on the effectiveness of the prevention, screening and treatment options for PPD, and offer up-to-date, evidence-based clinical practice guidelines for PPD management.

## Method

The evidence-based guidelines were developed by the Guidelines Development Group (GDG) of Riseup-PPD Cost Action based on the World Health Organization (WHO) manual for developing guidelines for clinical practice.^12^ The WHO Handbook is one of the reference manuals for the implementation of a clinical guide. Following the WHO recommendations, this group was multidisciplinary, formed by a group of 14 specialists in the field of peripartum mental health, from different fields of expertise (psychiatry, psychology, pharmacology, obstetrics, ethics, social work, information science), and also including a patient representative association and members from 12 different countries.

### Literature search

Ten patient/population, intervention, comparison and outcomes (PICO) questions were developed (see Supplementary Material 1). Six search strategies were developed to address the PICO questions. In the situation where there was an umbrella review available (and with one of the GDG members as an author), the evidence of that review was analysed to develop the recommendations. If this umbrella review was out of date, the GDG conducted an additional search for new systematic reviews. If there was no umbrella review available, a new search was performed. All searches were performed in five databases [CINAHL (Ebsco), the Cochrane Database of Systematic Reviews, Medline (Ebsco), PsycInfo (Ebsco) and Web of Science (Clarivate)], from 2010 until 2023, using title/abstract keywords and controlled vocabulary, as appropriate, in English. Search strategies are available in Supplementary Material 1.

### Study selection

Evidence for inclusion was limited to systematic reviews and meta-analyses. Study selection and data extraction were performed by at least two authors, with conflicts over inclusion adjudicated by a third author. Search strategies and PRISMA diagrams for each question are available in the appendix to the published guidelines. A quality assessment was performed using the AMSTAR 2 tool.^13^

### Recommendation development

The quality of the evidence was assessed using the GRADE system’s four levels (high, moderate, low and very low).^14^ The strength of the recommendations, noted as strong or weak,^14^ or no recommendation, is also reported in each recommendation. The recommendations for reporting evidence suggested by the AGREE II Checklist^15^ were followed. To develop each recommendation, the members of the GDG responsible for each PICO question presented the evidence that was retrieved and analysed and their recommendation, which were discussed within the GDG. The decisions were based upon consensus regarding the recommendations. In the situation where there was no consensus, the group proceeded with a voting system.

## Results

In total, 4539 studies were identified, 2873 were screened and 145 systematic reviews were included in the final analysis and used to develop the guidelines (available in Supplementary Material 2). The current document summarises the key questions and the recommendations from the ‘Evidence-based Clinical Practice Guidelines for Prevention, Screening and Treatment of Peripartum Depression’. Further background information and the supporting evidence for each recommendation can be found in the full version of the guidelines available from https://www.riseupppd18138.com/clinical-practice-guidelines.html.

**Fig. 1:**
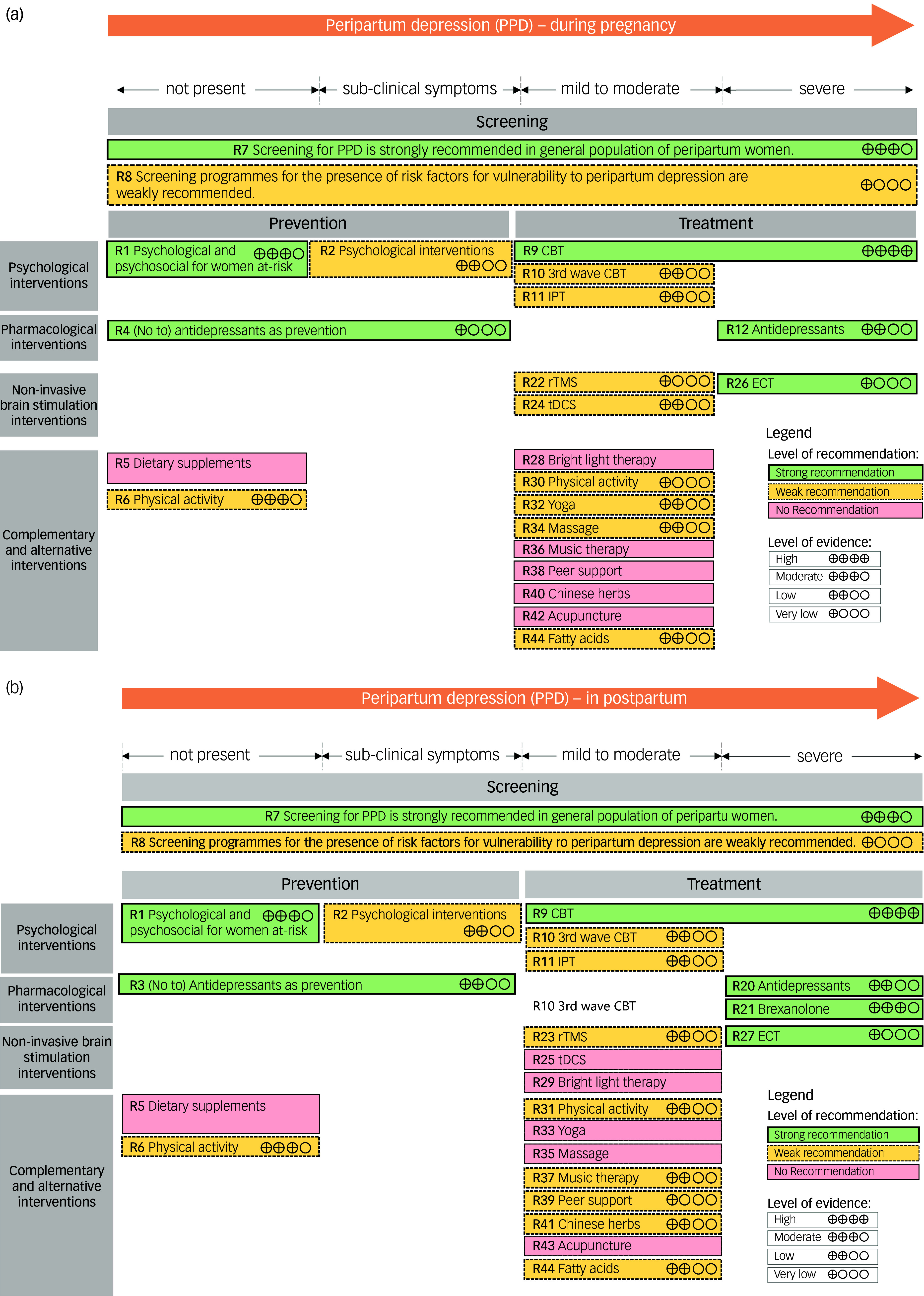
(a) Overview of the clinical recommendations for interventions during pregnancy. (b) Overview of the clinical recommendations for interventions in the postpartum period. CBT, cognitive–behavioural therapy; IPT, interpersonal therapy; rTMS, repetitive transcranial magnetic stimulation; tDCS, transcranial direct current stimulation; ECT, electroconvulsive therapy.

**Fig. 2:**
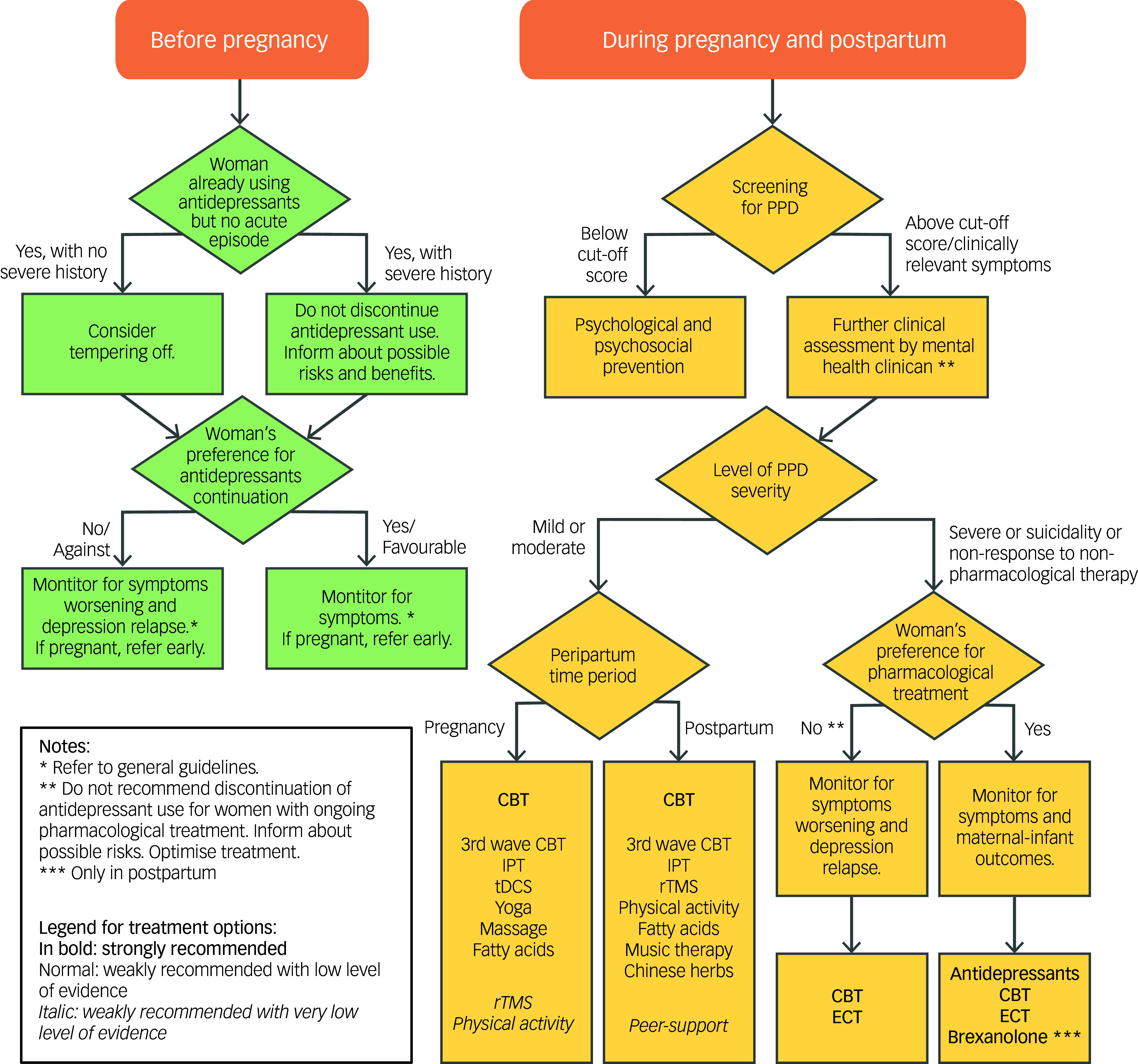
Clinical pathway for managing peripartum depression (PPD) in clinical settings. CBT, cognitive–behavioural therapy; IPT, interpersonal therapy; tDCS, transcranial direct current stimulation; rTMS, repetitive transcranial magnetic stimulation; ECT, electroconvulsive therapy.

Overview of the clinical practice recommendations for interventions during pregnancy and postpartum are presented in Figs. 1(a) and 1(b). Also, a decision tree with clinical pathway for managing PPD in a clinical setting is offered in Fig. 2.

### Prevention of PPD

#### Are interventions effective in preventing PPD among women from the general population, non-depressed, with no known risks or not identified as at risk for developing PPD?

The effectiveness of several interventions was examined: psychological and psychosocial interventions, antidepressant medication, dietary supplements and physical activity. Pregnant and postpartum women identified as at risk for developing PPD should receive psychological and psychosocial interventions for the prevention of PPD. Pregnant and postpartum women with no known risks for developing PPD could benefit from receiving psychological and psychosocial interventions for the prevention of PPD. Associated with efficacy studies, cost measures such as maternal/parental outcomes (e.g. morbidity, health-related quality of life, health services use, return to work), infant/child outcomes (e.g. preterm birth, infant mortality, vaccination, neurodevelopmental, socio-emotional and behavioural problems, school performance) and parenting outcomes (e.g. maltreatment of children and intimate partner violence (IPV)) should determine whether such types of interventions are worthwhile to implement (Table 1). Some recommended types of such preventive interventions for women at risk and women with no known risks include interventions based on cognitive–behavioural therapy (CBT) and interpersonal therapy (IPT), postpartum professional-based home visits, midwifery redesigned postnatal care and postpartum lay-based telephone support.^16–18^

**Table 1:** Summary of recommendations for prevention of peripartum depression (PPD)

Recommendation	Strength of recommendation/Quality of evidence	
R1	Psychological and psychosocial interventions are strongly recommended for preventing PPD among pregnant and postpartum women at risk of developing PPD, as well as among pregnant and postpartum women with no symptoms and no known risk factors.	Strong ⊕⊕⊕◯
R2	Psychological interventions are weakly recommended to prevent PPD among women with subclinical depressive symptoms.	Weak ⊕⊕◯◯
R3	It is not recommended to use antidepressant medication for prevention of postpartum depression in women with previous depression.	Strong ⊕⊕◯◯
R4	It is not recommended to use antidepressants for prevention of depression in pregnancy.	Strong ⊕◯◯◯
R5	We have no recommendation on use of dietary supplements to prevent PPD.	No recommendation.
R6	Physical activity is weakly recommended to prevent PPD among pregnant and postpartum women from the general population.	Weak ⊕⊕⊕◯

Note: Levels of recommendation: strong/weak/no recommendation; levels of evidence: ++++ high, +++ moderate, ++ low, + very low.

Pregnant and postpartum women with subclinical PPD symptoms could benefit from psychological and psychosocial interventions for the prevention of PPD. Some recommended types of such interventions include interventions based on CBT and IPT and parent–infant interaction treatment.^17^

Concerning pharmacological intervention to prevent PPD, there is one Cochrane review that investigated if antidepressant initiation after delivery could prevent PPD. The review includes two randomised controlled trial (RCT) studies with 73 women. The relative risk for postpartum depression was 0.14 (95% CI: 0.02–1.07, *p* = 0.058) in the sertraline arm versus placebo, but there was no difference between nortriptyline and the placebo group. Because of low statistical power and scarce evidence, a conclusion on the effectiveness of antidepressants in the prevention of PPD cannot be made.

There is no available evidence about the efficacy of antidepressant initiation during pregnancy for prevention of depression during pregnancy. There is limited evidence to recommend that dietary intake influences the risk of PPD.^18–20^

Pregnant and postpartum women could benefit from physical activity for the prevention of PPD. Some recommended types of physical activity include stretching, Pilates and aerobics. In addition, at least 90 min of physical activity per week are recommended for prevention of PPD.^18,21^

### Screening for PPD

When examining the evidence of screening programmes in detecting and/or reducing PPD, eight systematic reviews were included, out of which two were meta-analyses. These systematic reviews had different criteria for inclusion, such as type of studies considered, period of screening (during pregnancy and/or postpartum period), type of setting, delivery agents, provision of dedicated training for the involved professionals and enhanced care combined to screening. The instrument most often used was the Edinburgh Postnatal Depression Scale.^22^ Screening both in mid- to late pregnancy and between 2 and 3 months postpartum was reported. The scales used could be filled out by the participants themselves, or together with a healthcare professional. As no comparison was performed between these alternatives, no clear recommendation on exact timing of screening or exactly which tool to use can be made based on this review.

Overall, the evidence suggests a reduction in PPD and improved uptake rates of referral interventions among peripartum women undergoing screening programmes in healthcare settings. The direct evidence showed that screening women for depression during pregnancy and/or in the postnatal period may reduce depressive symptoms and reduce the prevalence of depression at follow-up in a given population, particularly in the presence of additional interventions combined to screening (e.g. treatment protocols, care management, availability of personnel trained on screening and on providing PPD counselling and/or treatment) provided on-site (Table 2).

**Table 2:** Summary of recommendations for screening of peripartum depression (PPD)

Recommendation	Strength of recommendation/Quality of evidence	
R7	Screening programmes for depression during pregnancy and in the postpartum period are strongly recommended.	Strong ⊕⊕⊕◯
R8	Screening programmes for the presence of risk factors for vulnerability to PPD are weakly recommended.	Weak ⊕◯◯◯

Note: Levels of recommendation: strong/weak/no recommendation; levels of evidence: ++++ high, +++ moderate, ++ low, + very low.

The indirect evidence showed that screening instruments could identify pregnant and postpartum women who need further evaluation and may need treatment. Patient satisfaction with PPD screening programmes was high in the five studies evaluating this aspect.^23^ Two studies^24,25^ included in the systematic review by Waqas et al^23^ evaluated cost-effectiveness and found screening interventions to be cost-effective. PPD screening should only be offered with adequate systems in place to ensure that women with positive screening results are appropriately diagnosed and treated according to evidence-based guidelines, by trained healthcare providers, within a specified time period, or promptly referred to a setting that can provide that level of care. Evidence on cost-effectiveness and acceptability should encourage policymakers and clinicians to address the challenges of introducing universal screening for PPD. These include, at least, ensuring the training of clinicians involved, supporting the collaborative care model between primary care and mental health professionals, the availability of crisis intervention and the provision of appropriate treatment for women with a confirmed diagnosis of PPD.

#### Are screening programmes effective in detecting the presence of risk factors for vulnerability to depression?

We specifically examined whether screening programmes were effective in detecting the presence of risk factors for vulnerability to depression. For this question, we identified two systematic reviews: Felice et al^26^ focused on four RCTs on pregnant women screened by a psychosocial assessment tool, for example, ALPHA or ANRQ, including known risk factors for PPD. When compared to routine care, psychosocial assessments were found to be sensitive to detect risk factors associated with postpartum depression. Results from three studies reported no statistically significant differences in postpartum depression. One study did not report on depression outcomes. One study found higher referral rates to social services in the intervention group, while another showed higher social support postpartum. The level of evidence varied.

In the systematic review on screening for IPV by Feltner et al,^27^ five studies focusing on IPV screening among peripartum women showed lower IPV rates postpartum in the screening group. Fewer postpartum depressive symptoms in the intervention group were reported in some studies. It is important to note that evidence based solely on systematic reviews was scarce in this area. Further research to assess efficacy, feasibility, acceptability, preferences and cost-effectiveness of such high-risk assessment programmes is warranted.

### Treatment of PPD

#### Psychological treatment of PPD

##### Are psychological interventions effective in treating PPD?

We identified 15 systematic reviews.^28–42^ Fourteen were consistent in showing that psychological interventions were effective in treating depressive symptoms during pregnancy and postpartum compared to no treatment (Table 3). With respect to different psychological approaches, the evidence is strongly related to CBT. The evidence for effectiveness of CBT’s third-wave techniques (behavioural activation, mindfulness-based techniques and acceptance-commitment therapy (ACT)) was weak. Furthermore, the evidence of IPT effectiveness was also weak. In summary, psychological treatments, especially CBT, are effective in treating PPD and should be used.

**Table 3:** Summary of recommendations for psychological treatment of peripartum depression

Recommendation	Strength of recommendation/Quality of evidence	
R9	Cognitive–behavioural therapy (CBT) is strongly recommended for the treatment of depressive symptoms during pregnancy and postpartum.	Strong ⊕⊕⊕⊕
R10	Third-wave CBT therapies, including behavioural activation and mindfulness techniques, are weakly recommended for the treatment of depressive symptoms during pregnancy and postpartum.	Weak ⊕⊕◯◯
R11	Interpersonal therapy (IPT) is weakly recommended for the treatment of depressive symptoms during pregnancy and postpartum.	Weak ⊕⊕◯◯

Note: Levels of recommendation: strong/weak/no recommendation; levels of evidence: ++++ high, +++ moderate, ++ low, + very low.

#### Pharmacological treatment of PPD

##### Are pharmacological interventions effective in treating PPD?

With regards to the effectiveness of antidepressant medication in PPD, the evidence is limited as double-blinded placebo-controlled randomised drug trials are difficult to conduct in pregnant and breastfeeding women because of ethical considerations. However, a Cochrane review^43^ showed superiority of antidepressant medication compared to placebo especially in women with moderate-to-severe depression. There are numerous meta-analysis and systematic reviews investigating the safety of antidepressant medication use in pregnancy with regards to malformation risk and other outcomes in exposed children. The evidence is quite strong that antidepressants in general are not major teratogens. In different drugs, there were hints of a mildly increased malformation risk, but the causality is not clear and rather doubtful. However, there is sufficient evidence that untreated depression can have negative impacts on exposed children;^44^ therefore, the risk of not treating depression should always be discussed and weighed against potential effects of the medication.

Discontinuation of existing antidepressant treatment is not recommended in women with a history of severe and recurrent disorder because of increased risk of relapse.^45^ However, to reduce potential negative effects of the medication, women should be treated with monotherapy and the lowest effective dose, if possible. Regular therapeutic drug measurements (TDMs), that is, measurement of the antidepressant blood concentration at designated time intervals, can be helpful in achieving those goals of optimal dosing, as there is evidence of changes in pharmacokinetics during the three trimesters of pregnancy.^46^ As clinicians and affected individuals are often reluctant to increase dosages in pregnancy even if depressive symptoms are not sufficiently controlled, TDM can support informed shared decision-making. However, prospective studies to investigate if TDM in pregnancy leads also to an improvement in clinical outcome are still lacking. As most likely the depression of the mother alone increases the risk for pregnancy and birth complications, affected women should give birth in hospitals with specialised neonatal care units.^47^

The Food and Drug Administration (FDA) has licensed the first specific medication for PPD treatment, the neurosteroid brexanolone. The evidence of two meta-analyses shows that brexanolone is more effective than placebo and more rapidly acting than selective serotonin reuptake inhibitors (SSRIs) in treating PPD.^48,49^ To date, there is no evidence that specific antidepressants would be more effective in women in pregnancy and/or postpartum.

In summary, antidepressant medication should always be prescribed after careful assessment of the individual risk–benefit ratio, including previous history, current severity of symptoms, patients’ preference and availability of alternative treatments (Table 4).

**Table 4:** Summary of recommendations for pharmacological treatment of peripartum depression

Recommendation	Strength of recommendation/Quality of evidence	
R12	Antidepressant medication in pregnancy is strongly recommended, after careful consideration of individual risk–benefit ratio for each woman and her unborn child. The decision-making about antidepressant intervention should consider the history of depression recurrence and severity of symptoms, previous response to the intervention, and individual preference.	Strong ⊕⊕◯◯
R13	Women with severe and/or recurrent depression are strongly recommended not to discontinue antidepressant medication during pregnancy because of the increased risk of relapse.	Strong ⊕⊕◯◯
R14	It is strongly recommended to closely monitor women who discontinue antidepressants upon recognition of pregnancy for symptoms worsening and risk of relapse.	Strong
R15	It is strongly recommended that pregnant women should be treated with antidepressants at the lowest effective dose and possibly as monotherapy.	GPP
R16	It is recommended to carry on regular monitoring of drug levels and to aim for low effective drug level during gestation.	GPP
R17	It is recommended to avoid abrupt discontinuation of the antidepressant upon recognition of the pregnancy.	GPP
R18	It is strongly recommended that clinicians provide information to women about the possible risks of antidepressant exposure in pregnancy on maternal-child health versus the potential risks posed by maternal depression.	Strong ⊕⊕⊕◯
R19	It is strongly recommended that all women under antidepressant interventions and their offspring should be closely monitored during pregnancy, as well as delivery being in a specialised obstetric centre with neonatal intensive care unit.	Strong ⊕⊕⊕◯
R20	It is strongly recommended to treat depression during the postpartum period with antidepressant medication, after careful consideration of individual risk–benefit ratio for each woman and her child if breastfed. The decision-making about antidepressant intervention should consider the history of depression recurrence and severity in the woman, previous response to the intervention and individual preference, as well as the health condition of the breastfed child.	Strong ⊕⊕◯◯
R21	It is strongly recommended to use brexanolone for moderate-to-severe postpartum depression treatment if available and if accepted as a treatment option by the woman.	Strong ⊕⊕⊕◯

GPP, good practice point.

Note: Levels of recommendation: strong/weak/no recommendation; levels of evidence: ++++ high, +++ moderate, ++ low, + very low.

### Non-invasive brain stimulation interventions for PPD

Four types of non-invasive brain stimulation interventions were considered: repetitive transcranial magnetic stimulation (rTMS), transcranial electrical stimulation (tES), specifically transcranial direct current stimulation (tDCS), electroconvulsive therapy (ECT) and bright light therapy (BLT) (Table 5).

**Table 5:** Summary of recommendations for non-invasive brain stimulation interventions of peripartum depression

Type of intervention	Recommendation	Strength of recommendation/Quality of evidence	
**rTMS**	R22	rTMS is weakly recommended for the treatment of mild-to-moderate depressive symptoms during pregnancy.	Weak ⊕◯◯◯
	R23	rTMS is weakly recommended for the treatment of mild-to-moderate depressive symptoms in the postpartum period.	Weak ⊕⊕◯◯
**tDCS**	R24	tDCS is weakly recommended for the treatment of mild-to-moderate depressive symptoms in pregnant women.	Weak ⊕⊕◯◯
	R25	There is not enough evidence to make a recommendation regarding the use of tDCS in the treatment of depression for women postpartum.	No recommendation
**ECT**	R26	ECT is strongly recommended for the treatment of therapy-resistant or life-threatening severe depression in pregnant women. The treatment should take place under strict obstetrical monitoring.	Strong ⊕◯◯◯
	R27	ECT is strongly recommended for the treatment of therapy- resistant or life-threatening severe depression in the postpartum period.	Strong ⊕◯◯◯
**BLT**	R28	There is no evidence on the efficacy of BLT in pregnancy; therefore, we cannot make a recommendation.	No recommendation
	R29	There is no evidence on the efficacy of BLT postpartum; therefore, we cannot make a recommendation.	No recommendation

rTMS, repetitive transcranial magnetic stimulation; tDCS, transcranial direct current stimulation; ECT, electroconvulsive therapy; BLT, bright light therapy.

Note: Levels of recommendation: strong/weak/no recommendation; levels of evidence: ++++ high, +++ moderate, ++ low, + very low.

Despite the overall low quality of the evidence regarding the efficacy of ECT in pregnancy and the postpartum period, ECT is a relatively fast-acting option in severe cases of depression with no response to previous regular treatment or when in need of urgent treatment because of life-threatening situations. Monitoring for outcomes and administration in specialised hospitals is recommended.

#### Are non-invasive brain stimulation (NIBS) interventions effective in reducing depressive symptoms during pregnancy and in the postpartum period pre–post treatment or when compared with other intervention or no intervention?

Although the evidence for repetitive transcranial magnetic stimulation (rTMS) is promising, the literature shows very low to low evidence of effectiveness both as a stand-alone and coadjuvant treatment with pharmacotherapy in reducing PPD symptoms during pregnancy and postpartum. The low level of evidence is caused by the weak design of the clinical studies and the lack of adequately powered RCTs.^50–54^

During pregnancy, only half of the studies showed rTMS efficacy as an adjunctive treatment to medication in pre- to post-intervention analysis; response and remission rates ranged between 38.8% and 81.8% and 18.8% and 61.1%, respectively. However, this effect was not significantly different compared to sham control groups.^53^

In the postpartum period, rTMS efficacy results are promising. Hence, despite the heterogeneity of treatment protocols, the evidence is of higher quality with optimal information size and large effect sizes (standardised mean difference (SMD) = −1.02 (95% CI [−1.37 to −0.66], *I* ^2^ = 83%).^54^

Evidence on adverse effects for the foetus during pregnancy show no association with the intervention and, for the women, adverse effects reported were equivalent to those found in the general population.^53^ Considering the literature on the safety profile of rTMS in the general population,^56^ severe adverse effects are unlikely in the postpartum stage.

Acceptability and feasibility data suggest women’s openness to treatment both during pregnancy and postpartum,^53,54,57^ particularly when using shorter protocols such as theta burst stimulation.

Although the evidence for rTMS is still scarce for PPD, considering the effectiveness on depressive symptoms in the general population and the safety profile, our recommendations consider that if rTMS is accessible and available, it can be an alternative treatment for pregnant or postpartum women with mild-to-moderate depressive symptoms, according to their clinical condition, values and preferences.

There is some evidence showing the benefit of tES as a stand-alone or coadjuvant with psychotherapy in improving depressive symptoms during pregnancy. Within tES, tDCS is the most studied strategy, showing a highly tolerable and safe profile for women and the foetus. Surprisingly, in the postpartum period, there is only one case report available in the literature showing a positive effect of tDCS to improve depressive symptoms. Therefore, despite tDCS’s benign effects in the general population, there is not enough data to make a recommendation on the use of tES postpartum.

Because of ethical concerns, the literature on the efficacy of ECT in PPD during pregnancy is of low quality, with no RCTs available, relying only on case series and case studies.^53,58,59^ All articles report patients with severe PPD, presenting life-threatening conditions.

Adverse effects on the mother were reported in about 27–52.4% of patients and include vaginal bleeding, uterine contractions, abdominal and pelvic pain, miscarriage, preeclampsia and premature birth and spontaneous abortion when the treatment was delivered during the first trimester.^58–60^ Caesarean, premature labour and uterine contractions associated with treatment are the most common obstetric complications associated with ECT treatments delivered in later semesters.^53^ Moreover, neurocognitive assessment after ECT extracted from one single study^61^ suggests that the maximum number of ECT sessions before achieving cognitive decline should be nine.

Rates ranging between 27.7% and 57.1% of significant adverse outcomes to the foetus and the child are also reported. These figures warrant strict supervision of ECT during pregnancy, which should be held preferably in a hospital setting by experienced healthcare teams.

The literature on using ECT in the postpartum^53,62^ encompasses case reports and retrospective and observational studies, observing postpartum women in the context of severe psychiatric disorders, unresponsive to other treatments. A percentage of 28% of adverse events was reported and remission rates were between 76.9% and 92%.

Therefore, despite the potential for serious adverse effects and complications, particularly during pregnancy and delivery, the benefits of ECT seen in the clinical practice often outweigh these risks in urgent, life-threatening situations where conventional treatments have failed. While the GDG recognises the very low level of evidence, we acknowledge the clinical experience of our experts, strongly recommending ECT where it can be a lifesaving intervention, which is the case with life-threatening, therapy-resistant severe perinatal depression (PPD). Note, however, that ECT treatments, particularly during pregnancy, should be held in hospital settings close to a labour ward, by experienced healthcare teams including an obstetrician, and warranting strict supervision, including cardiotocography monitoring.

Finally, the literature on the efficacy of BLT in reducing depressive symptoms in pregnant women^16,36,63–65^ reports inconsistent findings and the quality of the evidence is very low given the reduced number of women and the moderate risk of bias of the studies. As for BLT postpartum, only one study is available observing the effect of improving depressive symptoms in postpartum women. Therefore, there is no evidence on the efficacy of BLT that allows us to provide recommendations either during pregnancy or the postpartum period.

### Complementary and alternative therapies (CATs) for PPD

We considered eight types of CATs for PPD: fatty acids, physical exercise, yoga, massage, peer support, acupuncture, Chinese herbs and music therapy (Table 6).

**Table 6:** Summary of recommendations for complementary and alternative treatment interventions for peripartum depression (PPD)

Type of intervention	Recommendation	Strength of recommendation/Quality of evidence	
**Physical activity**	R30	Physical activity is weakly recommended for the treatment of mild-to-moderate depressive symptoms in otherwise healthy pregnant women.	Weak ⊕◯◯◯
	R31	Low-to-moderate intensity physical activity is weakly recommended for the treatment of mild-to-moderate depressive symptoms postpartum as it might be beneficial, and no risks of adverse effects are reported.	Weak ⊕⊕◯◯
**Yoga**	R32	Yoga is weakly recommended for the treatment of mild-to-moderate depressive symptoms as it might be beneficial, and no risks of adverse effects are reported.	Weak ⊕⊕◯◯
	R33	There is no evidence on the efficacy of yoga postpartum; therefore, we cannot make a recommendation.	No recommendation
**Massage**	R34	Massage is weakly recommended for the treatment of mild-to-moderate depressive symptoms during pregnancy.	Weak ⊕⊕◯◯
	R35	There is no evidence on the efficacy of massage postpartum; therefore, we cannot make a recommendation.	No recommendation
**Music therapy**	R36	There is no evidence on the efficacy of music therapy during pregnancy; therefore, we cannot make a recommendation.	No recommendation
	R37	There is no information on the effectiveness of music therapy as a stand-alone treatment for PPD postpartum. Music therapy is weakly recommended as an additive intervention when combined with other interventions (such as psychological treatment, Chinese medicine or pharmacotherapy) for the treatment of mild-to-moderate depressive symptoms as it might be beneficial, and no risks of adverse effects are reported.	Weak ⊕⊕◯◯
**Peer support**	R38	There is no evidence on the efficacy of peer support in pregnancy; therefore, we cannot make a recommendation.	No recommendation
	R39	There is no evidence to support a recommendation regarding traditional face-to-face peer support. Technology-based peer support is weakly recommended for the treatment of mild-to-moderate depressive symptoms in the postpartum period.	Weak ⊕◯◯◯
**Chinese herbs**	R40	There is no evidence on the efficacy of the use of Chinese herbs in pregnancy; therefore, we cannot make a recommendation.	No recommendation
	R41	Chinese herbs alone and in combination are weakly recommended for the treatment of mild-to-moderate depressive symptoms in the postpartum period.	Weak ⊕⊕◯◯
**Acupuncture**	R42	There is no evidence on the efficacy of the use of acupuncture in pregnancy; therefore, we cannot make a recommendation.	No recommendation
	R43	There is no evidence on the efficacy of the use of acupuncture in postpartum; therefore, we cannot make a recommendation.	No recommendation
**Fatty acids**	R44	Fatty acids are weakly recommended for the treatment of mild-to-moderate depressive symptoms in pregnancy and in the postpartum period.	Weak ⊕⊕◯◯

Note: Levels of recommendation: strong/weak/no recommendation; levels of evidence: ++++ high, +++ moderate, ++ low, + very low.

#### Are CATs effective in reducing depressive symptoms during pregnancy and in the postpartum period pre–post treatment or when compared with other interventions or no intervention?

Overall, the quality of the current evidence about the effectiveness of fatty acids in reducing depressive symptoms in pregnant and postpartum women is low to moderate, given its inconsistency and risk of bias. However, the direction of findings suggests their potential effect in mild-to-moderate depressive symptoms, when compared to placebo, but not in severe symptoms. Adverse effects associated with fatty acids are likely to be trivial.^66–69^

Physical exercise for pregnant women with depressive symptoms encompasses low-to-moderate or moderate-to-vigorous dancing, walking, aerobic exercise, stretching and relaxation. Although the available literature on the efficacy of physical exercise on depressive symptoms during pregnancy is of low quality, it shows a beneficial effect.^72^ Furthermore, whereas adverse effects of exercise interventions in pregnancy are absent for healthy pregnant women, physical exercise is counter indicated in pregnant women with several health conditions. The American College of Obstetricians and Gynecologists (ACOG) advises against aerobic exercise for pregnant women with certain conditions, such as hemodynamically significant heart disease, restrictive lung disease, incompetent cervix/cerclage, risk of premature labour in multiple gestations, persistent bleeding in the second or third trimester, placenta praevia, ruptured membranes and pregnancy-induced hypertension. In addition, the ACOG^11^ suggests that aerobic exercise may be inappropriate for pregnant women with severe anaemia, unassessed maternal cardiac arrhythmia, chronic bronchitis, extreme obesity, very low body weight (body mass index <12), lack of physical activity, intrauterine growth restriction, uncontrolled hypertension/preeclampsia, orthopaedic limitations, uncontrolled type II diabetes, seizure disorders, thyroid disease and a history of significant cigarette smoking. The ACOG also highlights specific warning signs, such as vaginal bleeding, shortness of breath before exertion, dizziness, headache, chest pain, muscle weakness, calf pain or swelling (which may indicate thrombophlebitis), signs of preterm labour, reduced foetal movement and leakage of amniotic fluid, which should prompt the immediate cessation of exercise.^71^

In the postpartum period, physical exercise (group or individual aerobic, walking and pram walking, whole-body stretching sessions; in-person or through e-health systems) seems to be of benefit in reducing depressive symptoms. However, the heterogeneity of exercise interventions described in the literature results in some degree of uncertainty about the parameters for optimal results.

Despite the low quality of the evidence, the literature shows that yoga might be beneficial to improve depressive symptoms during pregnancy in physically healthy women^33,72^ with unlikely associated risks for the foetus/newborn. This recommendation is cautious, as the literature is unclear regarding symptoms’ severity and is based on low-quality evidence. In the postpartum period there is no evidence of the efficacy of yoga to improve depressive symptoms.^33,73,74^

Although the literature is of low quality, massage might be beneficial to improve depressive symptoms during pregnancy^70^ when it is applied by partners or therapists. Given there are currently no studies on the effect of massage in the postpartum period, no recommendation is possible.

To date, there is only one study observing the effects of peer support in PPD during pregnancy and therefore a recommendation is not possible. Postpartum, interventions are recommended to be provided by health professionals (mediators) or trained peers who have similarities to the pregnant women and have personal experience of previous PPD. No adverse effects are expected, and rates of satisfaction are expected to be high. Interventions can be delivered individually or in a group, or a combination of the two, and those that are technology-based seem to be more consistent. However, while technology increases accessibility it might also increase inequality with those who do not have the resources or the willingness to use digital alternatives.^75,76^

The available literature on the use of acupuncture for PPD is inconsistent.^36,64^ Moreover, electro-acupuncture shows transient adverse effects.^77^ No recommendation for the use of acupuncture is possible at the current stage.

There is no current literature available on the effect of Chinese herbs in reducing depressive symptoms in pregnancy and a recommendation cannot be presented at this stage.

In postpartum depression, Shuganjieyu capsules compared to regular antidepressants (e.g. citalopram, paroxetine, fluoxetine, sertraline) or in combination (e.g. Chai Hu Shu Gan San and fluoxetine) seem to be promising.^74,78–80^ Although no significant adverse effects were reported, the safety of Chinese herbs for the newborn through breastfeeding is unknown. Given the low quality of the included studies, we weakly recommend Chinese herbs in the postpartum period. Moreover, special caution for St. John’s wort should be made as it limits the effectiveness of many prescription medicines and combining it with certain antidepressants can lead to a potentially life-threatening condition.^81^

Currently, the effect of music therapy as stand-alone treatment in depressive symptoms in pregnant women is still unknown and a recommendation is not possible.

Postpartum, music therapy (e.g. light music, pure music, lullaby) combined with psychological treatment and pharmacotherapy can improve depressive symptoms, insight, pain, sleep, satisfaction and maternal bonding with no adverse side effects expected.^82^

## Discussion

This paper summarises the 44 recommendations from the Riseup-PPD Evidence-based Guidelines for Prevention, Screening, and Treatment of Peripartum Depression, based on an extensive literature review. To our knowledge, these are the first evidence-based guidelines offering recommendations specifically for the management of PPD, representing a substantial advancement towards improved and high-quality peripartum mental health care. Although previous guidelines have addressed peripartum mental health,^8,10^ they have not focused specifically on PPD. Furthermore, the majority of previous guidelines^9^ exhibited a high level of bias and provided limited information on prevention, and treatment was mostly focused on pharmacological and less on psychological treatments with even fewer references to alternative options, such as non-invasive brain stimulation and complementary therapies. Because PPD is highly prevalent disorder in the peripartum period, and subject to specific prevention, screening and treatment interventions, it is worth addressing this topic in an exclusive way.^83^ This is specifically needed given the recent review showing several specificities of PPD compared to MDD, including the symptom presentation, risk factors and other clinical features.^2^ This guide provides a clear path for policymakers. Based on the evidence presented in this guide, several policies should be implemented to improve healthcare for peripartum women. To prevent PPD, protocols should be established to discourage the use of antidepressants as a preventive measure and encourage the participation of professionals in psychological and psychosocial interventions. In addition, population screening for PPD should be conducted for all peripartum women. Currently, the available screening tools are easy to administer and do not incur additional costs as they are openly accessible. This facilitates their integration into healthcare systems. It is important to implement screening practices at some point during pregnancy and within the first months postpartum, and have clear care pathways in place in case of positive screening. Consequently, policymakers should recognise the urgent necessity for primary care and maternal health professionals to receive the necessary training and resources to perform screening procedures. Furthermore, it is essential to establish clear and accessible referral pathways among primary care, maternal health and mental health services. High patient acceptability and satisfaction, cost-effectiveness and a reduction of rates of depression should be considered among the pros of universal screening. On the other hand, some increase in visits is needed focusing on the screening and follow-up (which is counter-balanced in studies by visits needed for treatment and general higher pathology among mothers and children after a full depressive episode that is not timely treated), and a possible transient anxiety after a positive screen among those not suffering from depression could be considered among negative side-effects of universal screening.

Healthcare professionals, researchers and other stakeholders should bear in mind that different barriers are preventing peripartum women seeking help for PPD, including psychosocial factors, such as stigma or lack of knowledge, structural factors, such as lack of information about services, and instrumental factors, such as financial restraints.^84,85^ Healthcare systems should be wisely organised to overcome these obstacles.

Several suggestions for future studies arose from the conducted reviews. One notable bias observed in the studies was the absence of randomised controlled studies with universal samples. Studies often focus on specific populations, neglecting typical peripartum women. Furthermore, it is important to note the lack of comparative studies, such as those between psychological therapies or between psychological and pharmacological therapies. Research on the mechanisms that explain why preventive interventions or treatments work (response to treatment) remains scarce. In addition, the long-term outcomes of treatments are still unknown, and further studies are needed. We focused exclusively on women in this study, even though there is growing evidence that fathers can also experience PPD.^86,87^ Unfortunately, it was not possible to include fathers in our recommendations because of the significant lack of research on effective interventions for them, highlighting a crucial area for future studies.

Despite these merits, several limitations of this work should be considered. First, the evidence that was collected is limited to systematic reviews and meta-analyses. Although this option allows for the collection of a high degree of evidence, it has the disadvantage of excluding individual papers, namely RCTs, that have not been included yet in a systematic review because of their recency, or for other reasons. For this reason, there may be individual studies that offer relevant information on the efficacy of some interventions that were excluded from our analysis. With updated literature on novel effective approaches in prevention, screening and treatment, the recommendations should be updated, also. Given the rapid increase in new studies, we suggest that the Clinical Practice Guidelines should be updated in 5 years. Also, more studies are needed in respect to socioeconomic context, such as the most recent review on the effectiveness of IPT in low- and middle-income countries.^88^ Given that most of the original studies come from high-income countries, but that mental health care services differ between high-income and low- to middle-income countries,^89^ further efforts need to be undertaken to implement these guidelines in different socioeconomic contexts and healthcare systems.

Since women constitute more than 51% of the European population, their health – both physical and mental – should matter to Europe and its key decisionmakers. These guidelines offer 44 recommendations on prevention, screening and treatment of PPD that should direct PPD management based on up-to-date evidence. It is important to make sure these European guidelines will be adopted at a national level to support the lives of the mothers of the 4 million babies being born every year in Europe.

## Supporting information

Radoš et al. supplementary material 1 Radoš et al. supplementary material

Radoš et al. supplementary material 2 Radoš et al. supplementary material

Radoš et al. supplementary material 3 Radoš et al. supplementary material

## Data Availability

The data that support the findings of this study are available in the supporting information file Supplementary Material 2.

## Supplementary material

The supplementary material can be found online at https://doi.org/10.1192/bjp.2025.43
