# Supplementary material for: Evidence-based clinical practice guidelines for prevention, screening and treatment of peripartum depression
Source: Br J Psychiatry. 2025 Jun 26;227(5):798–809. doi: 10.1192/bjp.2025.43 (PMC12550661; doi:10.1192/bjp.2025.43)

Appendix: Search strategies and PRISMA diagrams

Prevention of PPD

Are interventions in the form of psychological and psychosocial interventions effective in preventing PPD among women from the general population, non-depressed, with no known risks or not identified as at-risk for developing PPD?

Are interventions in the form of psychological and psychosocial interventions effective in preventing PPD among women with sub-clinical depressive symptoms?

Are interventions in the form of dietary supplements effective in preventing PPD?

Are interventions in the form of physical activity effective in preventing PPD?

These recommendations are based on the recent umbrella review, Motrico et al. (2023).

That study identified 19 relevant RCT-based studies, which were imported into Covidence and evaluated and extracted using this project's criteria. However, Motrico et al. also identified an additional 13 non-RCT-based studies, which would otherwise have been included apart from the excluded trial type. As such studies would not have been excluded according to *this* project's criteria, they were also imported into Covidence and screened. Thus, a total of 32 studies were screened.

Of these, 31 were deemed relevant based on the project's criteria. Six studies were excluded at the full-text level, leading to 25 studies being extracted.

During the data extraction phase five studies were excluded from the recommendations: three studies (Lin et al., 2018; McCurdy et al., 2017; Zhu et al., 2021) were excluded since they did not have enough information to differentiate between prevention and treatment of PPD, and, therefore, it was impossible to extract the data for the prevention recommendations. One study (Adina et al., 2022) was excluded since during the data extraction phase it became clear that most baseline depressive symptoms were indicative of depressive symptoms (10 or above on the EPDS) which means they were treatment and not preventive interventions. One more study (Miller et al., 2013) was excluded as during the data extraction phase it became clear that it included only two studies, one of which included girls under the age of 18. These studies were, therefore, not included in the data extraction table.

The team next sorted the studies by type of intervention (i.e., psychological and psychosocial interventions, exercise interventions, dietary supplements as intervention and pharmacological interventions) and recommendations were provided separately for each type of intervention. Studies which included a variety of interventions were included if results were presented separately for each type of intervention. Based on this, three further studies were excluded. Two studies (Saad et al., 2021; Zhou et al., 2022) were excluded as they included a mixture of interventions with results that were not specific to a certain type of intervention and the team could not extract data that were specific to the types of interventions. One study (Sasaki et al., 2021) was excluded as while it was the only study on maternal-infant interventions, it was based on only two studies and its quality was low, therefore making it very difficult to provide sufficient recommendations.

Prior umbrella review

Motrico, E., Bina, R., Kassianos, A. P., Le, H.-N., Mateus, V., Oztekin, D., Rodriguez- Muñoz, M. F., Moreno-Peral, P., & Conejo-Cerón, S. (2023). Effectiveness of interventions to prevent perinatal depression: An umbrella review of systematic reviews and meta-analysis. *General Hospital Psychiatry*, *82*, 47-61. https://doi.org/10.1016/j.genhosppsych.2023.03.007

PRISMA diagram. Prevention of PPD

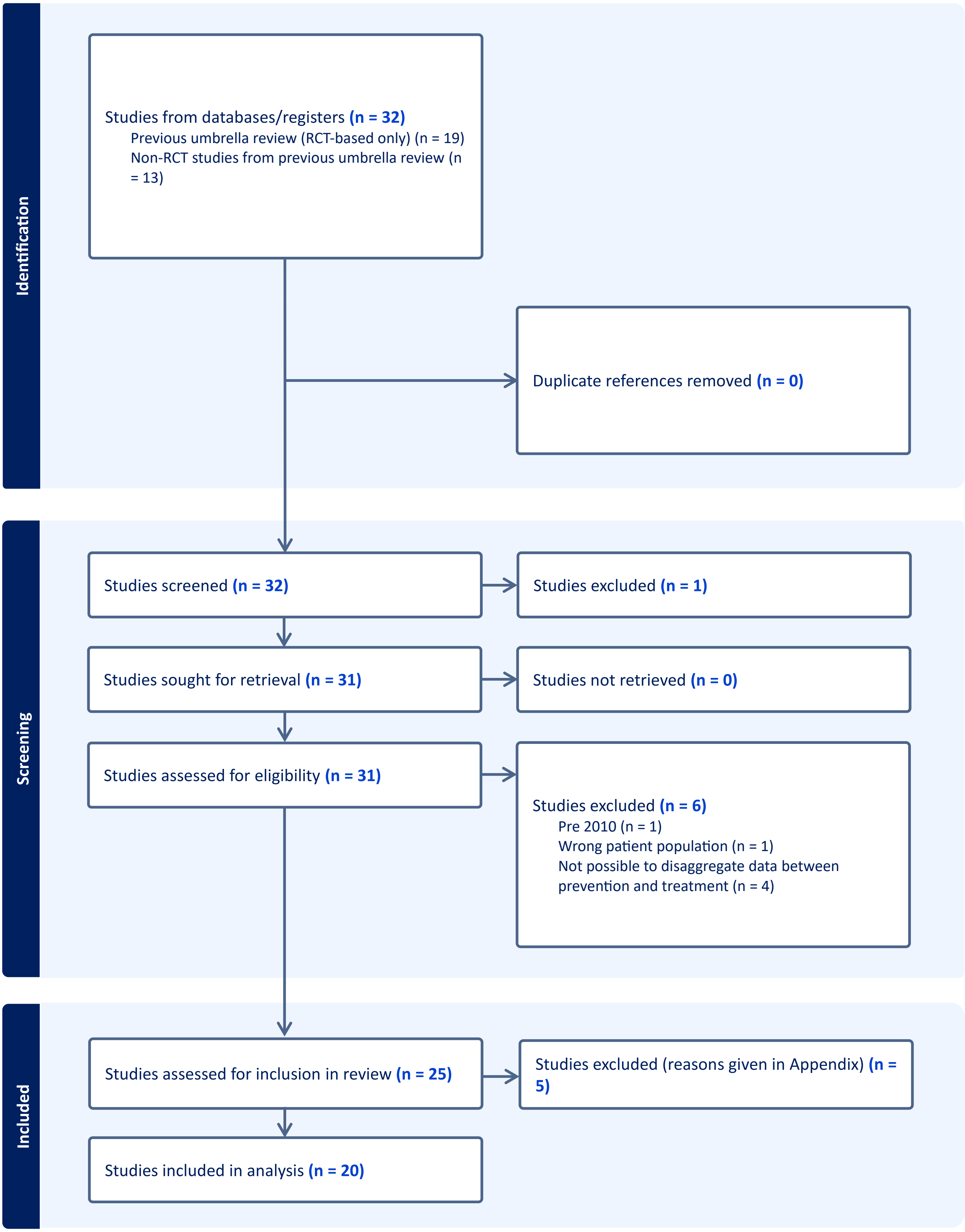

Screening for PPD

Are screening programmes effective in detecting and/or reducing peripartum depressive symptoms?

Are screening programmes effective in detecting women at high-risk and reducing peripartum depressive symptoms?

These recommendations are based on performing a search appropriate for an original umbrella review for this project.

A comprehensive and systematic search strategy appropriate for an umbrella review was constructed by adapting strategies in previous studies on PPD and screening coupled with input from the project team. This was trialled in a sample database, edited based on comments from the team, and implemented by the information specialist. CINAHL (Ebsco), the Cochrane Database of Systematic Reviews, Medline (Ebsco), PsycInfo (Ebsco) and Web of Science (Clarivate) were all searched from 2010 until 17 November 2022.

This resulted in a total of 3108 studies which were uploaded into Covidence for screening; after deduplication, 1808 were screened at title/abstract level for relevance by a combination of two members of the team. This led to 55 studies being screened at full-text level and ultimately a small number of relevant studies (n=15) being included. These were then data-extracted by two members of the team.

Four studies were excluded during the data extraction phase. One study (El-Den et al., 2015), focusing on screening acceptability and one study (Martínez-Borba et al., 2018), focusing on integrating communication technologies in screening, were excluded since they did not provide information on the impact of the described approach on perinatal depressive symptoms (specified primary outcome). One study (Lassi et al., 2014) providing evidence that intimate partner violence is related to an increased risk of depression or unintended pregnancy among the victims; it was excluded as it did not provide information on depression during the perinatal period (specified primary outcome). Lastly, one study (Murthy et al., 2021), on the screening for postpartum mood and anxiety disorders among caregivers of infants hospitalised in the neonatal intensive care unit, was excluded due to the focus on a high-risk population, which could not be compared with that of the other included studies.

Although not a part of the search criteria, only studies in English were included. This is recognised as a possible limitation of the current study.

An example search in Medline is shown below.

Medline (Ebsco) search

Limits: 2010 onwards; Peer-reviewed; Human; Publication Type: Meta Analysis, Systematic Review

(MH "Depression, Postpartum" OR (TI(antenatal OR "ante natal" OR antepartum OR "ante partum" OR birth* OR breastfe* OR childbearing OR childbirth* OR labor OR labour OR lactat* OR matern* OR mother* OR parturition OR perinatal OR peripartum OR postnatal OR "post natal" OR postpartum OR "post partum" OR pregnan* OR prenatal OR "pre natal") OR AB(antenatal OR "ante natal" OR antepartum OR "ante partum" OR birth* OR breastfe* OR childbearing OR childbirth* OR labor OR labour OR lactat* OR matern* OR mother* OR parturition OR perinatal OR peripartum OR postnatal OR "post natal" OR postpartum OR "post partum" OR pregnan* OR prenatal OR "pre natal")) AND (TI(depress*) OR AB(depress*)))

AND

(MH "Mass Screening" OR MH "Neonatal Screening" OR TI(((at OR high*) N2 risk*) OR aware* OR "check list*" OR checklist* OR detect* OR diagnos* OR (early N2 identif*) OR intervention* OR predict* OR prevent* OR program* OR psychodiagnos* OR questionnaire* OR scale* OR screen*) OR AB(((at OR high*) N2 risk*) OR aware* OR "check list*" OR checklist* OR detect* OR diagnos* OR (early N2 identif*) OR intervention* OR predict* OR prevent* OR program* OR psychodiagnos* OR questionnaire* OR scale* OR screen*))

PRISMA diagram. Screening of PPD

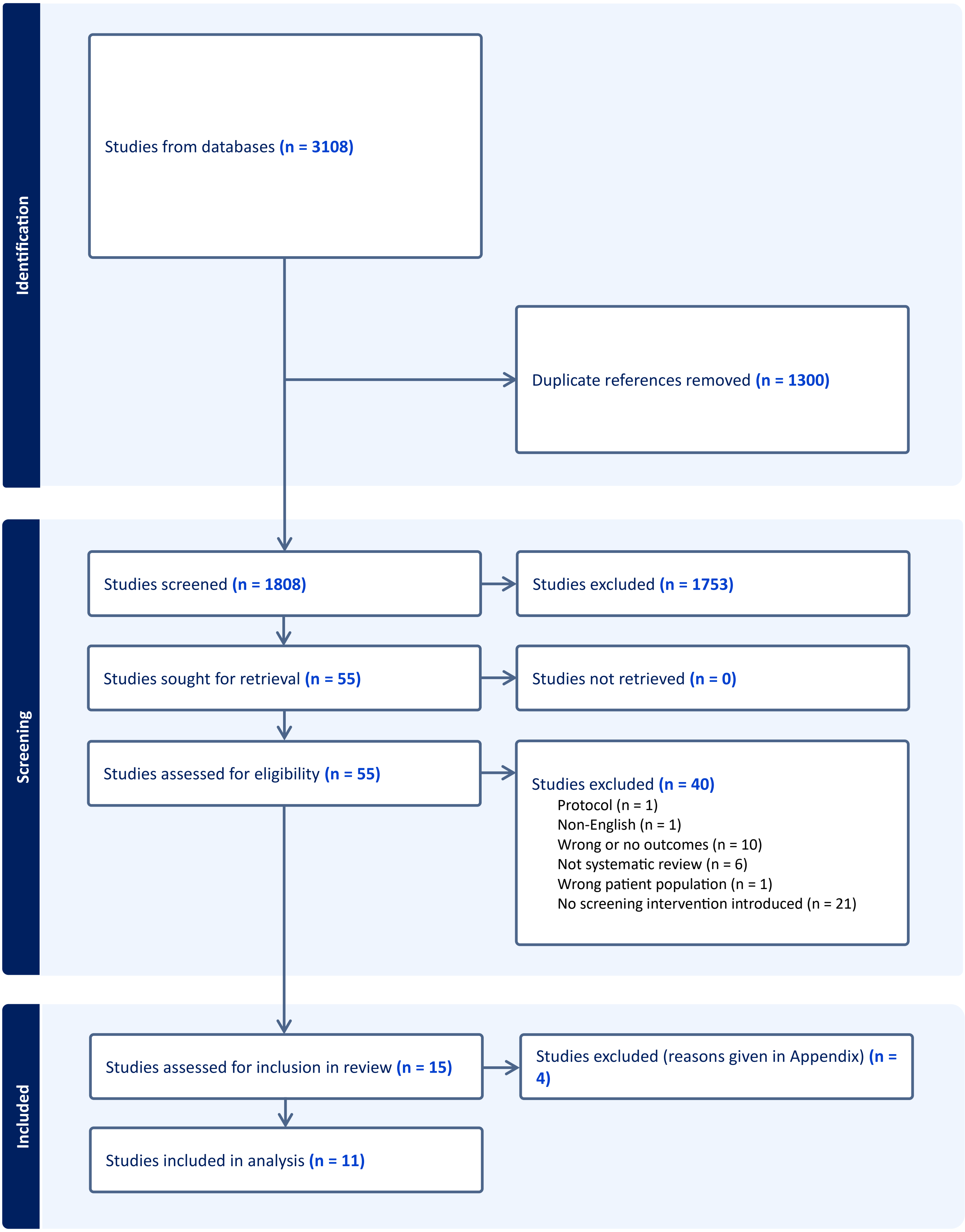

Treatment of PPD: Psychological interventions

Are psychological interventions effective in treating PPD?

These recommendations are based in part on the recent umbrella review, Branquinho et al. (2021). Due to the age of that study, an additional search was undertaken to find recent relevant results published after the search for that study was completed.

Branquinho et al. identified seven relevant studies, which were imported into Covidence and evaluated using this project's criteria.

To find recent results, a comprehensive and systematic search strategy appropriate for an umbrella review was constructed by adapting strategies in previous studies on PPD and psychological treatment (including Branquinho et al.) coupled with input from the project team. This was trialled in a sample database, edited based on comments from the team, and implemented by the information specialist. CINAHL (Ebsco), the Cochrane Database of Systematic Reviews, Medline (Ebsco), PsycInfo (Ebsco) and Web of Science (Clarivate) were all searched from 2020 until 16 January 2023. A total of 388 studies were identified.

An example search in Web of Science is shown below.

Combining the prior results with this new search resulted in a total of 395 studies; after deduplication, a total of 261 were screened at title/abstract level in Covidence for relevance by a combination of two members of the team. This led to 60 studies being screened at full-text level and ultimately a small number of relevant studies (n=17) data-extracted by two members of the team. Of these, two studies were rejected as they did not disaggregate data between treatment and prevention, leading to the final amount included in the analysis (n=15).

Although not a part of the search criteria, only studies in English were included. This is recognised as a possible limitation of the current study.

Prior umbrella review

Branquinho, M., Rodriguez-Muñoz, M. d. l. F., Maia, B. R., Marques, M., Matos, M., Osma, J., Moreno-Peral, P., Conejo-Cerón, S., Fonseca, A., & Vousoura, E. (2021). Effectiveness of psychological interventions in the treatment of perinatal depression: A systematic review of systematic reviews and meta-analyses. Journal of Affective Disorders, 291, 294-306. https://doi.org/10.1016/j.jad.2021.05.010

Web of Science search

Limits: Run in Topic field only, 2020 onwards, Review Articles

(((antenatal OR "ante natal" OR antepartum OR "ante partum" OR birth* OR breastfe* OR childbearing OR childbirth* OR labor OR labour OR lactat* OR matern* OR mother* OR parturition OR perinatal OR peripartum OR postnatal OR "post natal" OR postpartum OR "post partum" OR pregnan* OR prenatal OR "pre natal") AND (depress*)))

AND

(counseling OR counselling OR psychotherap* OR ((psych* OR supportive) NEAR/5 (intervention* OR treat* OR therap*)))

AND

(((evidence OR gap) NEAR/2 map*) OR EGM OR "meta analy*" OR metaanaly* OR "research synthes*" OR ((systematic OR rapid OR realist OR impact) NEAR/2 (review* OR assessment* OR stud*)))

PRISMA diagram. Treatment of PPD: Psychological interventions

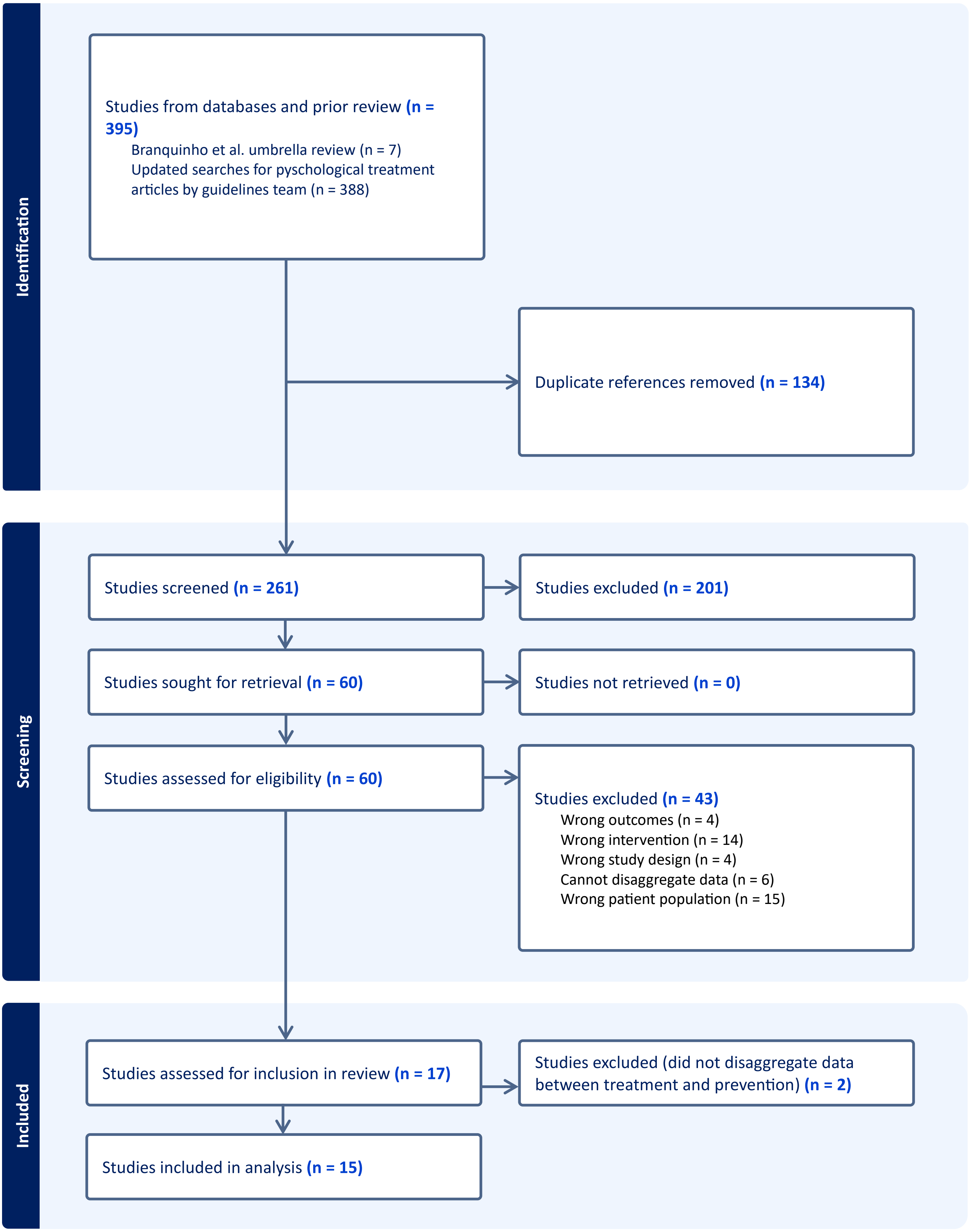

Treatment of PPD: Pharmacological interventions

Are pharmacological interventions effective in treating PPD?

These recommendations are based on performing a search appropriate for an original umbrella review for this project.

A comprehensive and systematic search strategy appropriate for an umbrella review was constructed by adapting strategies in previous studies on PPD and antidepressants coupled with input from the project team. This was trialled in a sample database, edited based on comments from the team, and implemented by the information specialist. CINAHL (Ebsco), the Cochrane Database of Systematic Reviews, Medline (Ebsco), PsycInfo (Ebsco) and Web of Science (Clarivate) were all searched from 2010 until 3 January 2023.

This resulted in a total of 610 studies which were uploaded into Covidence for screening; after deduplication, a total of 385 were screened at title/abstract level for relevance by a combination of two members of the team. This led to 113 studies being screened at full-text level and ultimately a relatively large number of relevant studies (n=76) being included for possible extraction.

The team next sorted these studies by outcomes (for example, all studies looking at autism in children exposed to antidepressants were put together in a group). Then, in each group they selected and extracted data for the most recent study. If the quality of this most recent study was deemed poor, they extracted an additional study within the same group that had sounder methodology. For studies looking at specific antidepressant substances, they extracted all relevant studies (please note that most studies look at antidepressants as a class; the team considered it important to extract data for each substance when available).

In total, n=38 studies were data-extracted by two members of the team and included in the analysis.

Although not a part of the search criteria, only studies in English were included. This is recognised as a possible limitation of the current study.

An example search in CINAHL is shown below.

CINAHL (Ebsco) search

Limits: 2010 onwards; Peer-reviewed; Human; Exclude MEDLINE; Publication Type: Meta Analysis, Meta Synthesis, Systematic Review

(MH "Depression, Postpartum" OR (TI(antenatal OR "ante natal" OR antepartum OR "ante partum" OR birth* OR breastfe* OR childbearing OR childbirth* OR labor OR labour OR lactat* OR matern* OR mother* OR parturition OR perinatal OR peripartum OR postnatal OR "post natal" OR postpartum OR "post partum" OR pregnan* OR prenatal OR "pre natal") OR AB(antenatal OR "ante natal" OR antepartum OR "ante partum" OR birth* OR breastfe* OR childbearing OR childbirth* OR labor OR labour OR lactat* OR matern* OR mother* OR parturition OR perinatal OR peripartum OR postnatal OR "post natal" OR postpartum OR "post partum" OR pregnan* OR prenatal OR "pre natal")) AND (TI(depress*) OR AB(depress*)))

AND

(MH "Antidepressive Agents+" OR TI(allopregnanolon* OR "anti depress*" OR antidepress* OR brexanolon* OR hormon* OR neurosteroid* OR pharmacolog* OR "SSRI*" OR "SNRI" OR ((reuptake OR uptake) N5 inhibitor*) OR tricyclic*) OR AB(allopregnanolon* OR "anti depress*" OR antidepress* OR brexanolon* OR hormon* OR neurosteroid* OR pharmacolog* OR "SSRI*" OR "SNRI" OR ((reuptake OR uptake) N5 inhibitor*) OR tricyclic*))

PRISMA diagram. Treatment of PPD: Pharmacological interventions

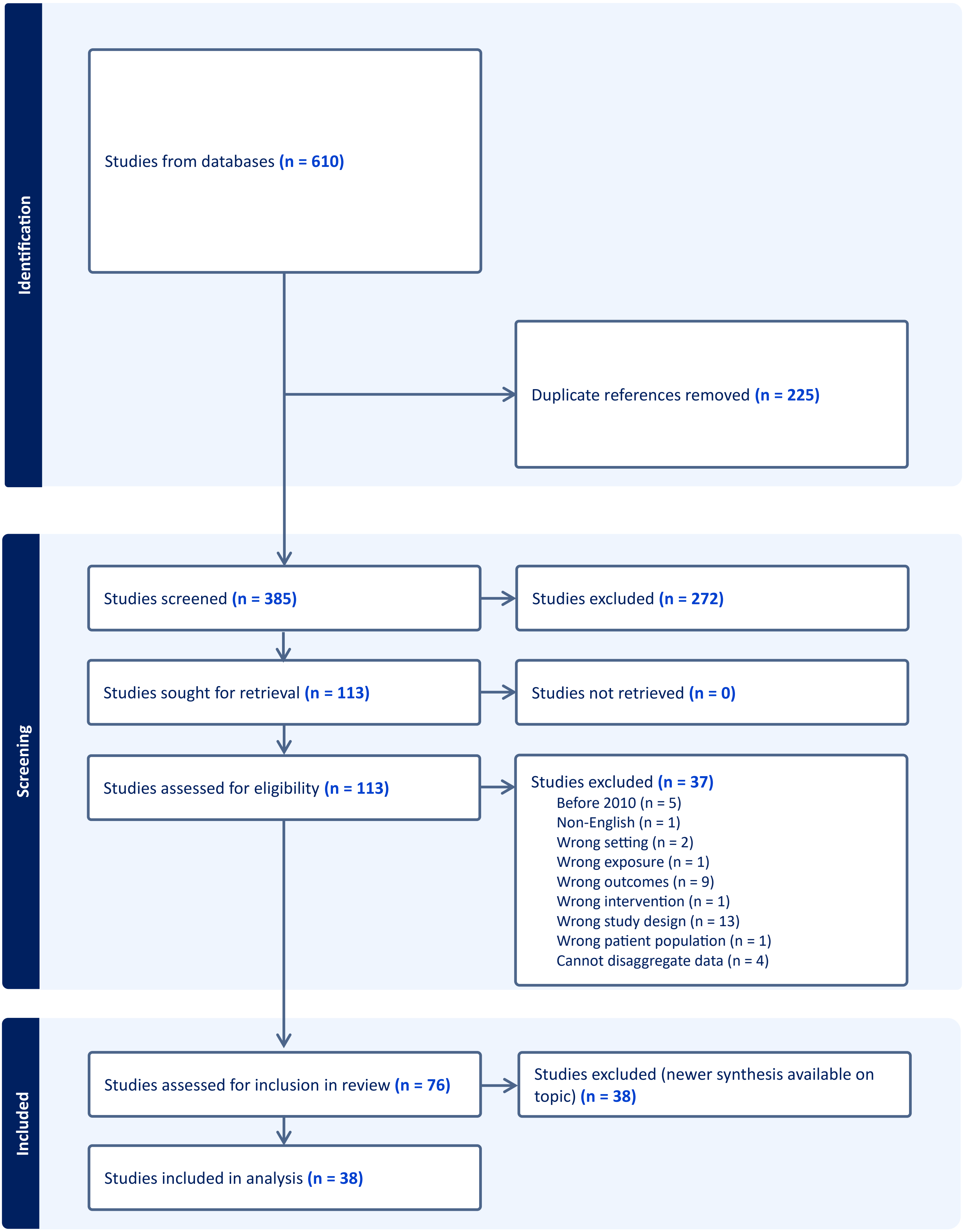

Treatment of PPD: Non-invasive brain stimulation interventions

Are non-invasive brain stimulation and bright light therapy interventions effective in treating PPD?

Treatment of PPD: Complementary and alternative treatments

Are complementary and alternative treatment interventions effective in treating PPD?

These recommendations are partly based on a forthcoming umbrella review, detailed in the protocol Dubreucq et al. (2022). For that review, AMED (Ebsco), CINAHL (Ebsco), Embase (Elsevier), Medline (Ovid), PsycInfo (Ovid) and Google Scholar were all searched from inception until 14 April 2021; an update was performed by the information professional for the original team on 23 January 2023.

The search for Dubreucq et al. identified 68 relevant studies for extraction in 2021, which were imported into Covidence to be evaluated using this project's criteria. 310 prospective results were identified by the 2023 update.

As non-invasive brain stimulation (NIBS) and bright light therapy (BLT) interventions were not part of the inclusion criteria for extraction in Dubreucq et al., a comprehensive and systematic search strategy appropriate for an umbrella review was constructed by adapting strategies in previous studies on PPD and NIBS/BLT coupled with input from the project team. This was trialled in a sample database, edited based on comments from the team, and implemented by the information specialist. CINAHL (Ebsco), the Cochrane Database of Systematic Reviews, Medline (Ebsco), PsycInfo (Ebsco) and Web of Science (Clarivate) were all searched from 2010 until 14 March 2023. A total of 103 studies were identified; after internal deduplication, 56 studies were screened at title/abstract level and 24 relevant to NIBS/BLT screened at the full-text level. After screening of full text, a small number of relevant studies (n=19) were deemed suitable for extraction.

Combining the original results from Dubreucq et al. on CAT, the new update, and the ones selected for extraction from the BLT and NIBS search resulted in a total of 62 studies included for extraction by two members of the team. One study was subsequently excluded from the analysis as it only included women from the general population without clinically assessed PPD, meaning a total of 61 reports are included in the analysis.

Although not a part of the search criteria, only studies in English and published after 2010 were included. This is recognised as a possible limitation of the current study.

Example searches in Embase for CAT and the Cochrane Database of Systematic Reviews for NIBS and BLT are shown below.

Prior umbrella review protocol

Dubreucq, J., Kamperman, A. M., Al-Maach, N., Bramer, W. M., Pacheco, F., Ganho-Avila, A., & Lambregtse-van den Berg, M. (2022). Examining the evidence on complementary and alternative therapies to treat peripartum depression in pregnant or postpartum women: study protocol for an umbrella review of systematic reviews and meta-analyses. BMJ Open, 12(11), e057327. https://doi.org/10.1136/bmjopen-2021-057327

Complementary and alternative therapies: Embase search (forthcoming umbrella review)

('perinatal depression'/exp OR 'peripartum depression'/de OR (depression/exp AND (pregnancy/exp OR 'pregnant woman'/de OR 'prenatal period'/de OR 'perinatal period'/de OR 'pregnancy complication'/de OR 'obstetric procedure'/exp)) OR ((depressi* OR psychiatr*) NEAR/3 (peripart* OR peri-part* OR perinatal* OR prenatal* OR pre-natal* OR postnatal* OR peri-natal* OR post-natal* OR pregnan* OR postpart* OR post-part* OR antepart* OR ante-part* OR antenatal* OR ante-natal* OR obstet* OR labor OR labour OR maternal* OR mother*)):ab,ti)

AND

('physical activity'/exp OR kinesiotherapy/exp OR exercise/exp OR massage/exp OR mindfulness/exp OR meditation/exp OR 'alternative medicine'/exp OR acupuncture/exp OR 'herbaceous agent'/de OR 'traditional medicine'/exp OR phototherapy/exp OR 'music therapy'/de OR supplementation/exp OR 'essential oil'/exp OR hypnosis/de OR 'body psychotherapy'/de OR telehealth/exp OR 'mobile application'/exp OR 'mobile phone'/exp OR

'peer group'/de OR 'acceptance and commitment therapy'/de OR 'systemic therapy'/de OR 'holistic care'/de OR 'couple therapy'/de OR 'fatty acid'/exp OR 'medicinal plant'/exp OR 'plant extract'/exp OR 'plant medicinal product'/de OR 'dietary supplement'/exp OR 'diet therapy'/exp OR vitamin/exp OR magnesium/de OR 'amino acid'/exp OR lipid/exp OR 'natural product'/de OR fish/de OR 'sea food'/exp OR ((physical* NEAR/3 activit*) OR kinesiotherap* OR kinesitherap* OR yoga OR pilates OR tai-chi OR exercis* OR massage* OR mindfulness* OR mind-body* OR meditation* OR homeopath* OR acupuncture* OR phototherap* OR light-therap* OR bright-light* OR supplement* OR essential-oil OR hypnosis OR hypnotherap* OR hypno*-therap* OR (body NEXT/1 (psychotherap* OR psycho-therap* OR orient*)) OR telehealth* OR telemedicine OR ehealth OR e-health OR m-health OR mhealth OR (mobile NEXT/1 (application OR app OR apps OR phone)) OR smartphone* OR smart-phone* OR (peer NEXT/1 (group* OR support*)) OR (acceptan* NEAR/3 commitment* NEAR/3 therap*) OR ((systemic) NEAR/3 therap*) OR ((couple OR partner OR relation*) NEAR/3 therap*) OR ((alternative OR complementar* OR traditional* OR herbal* OR folk* OR music* OR African* OR anthroposophic* OR bioresonance* OR aroma* OR Chinese* OR energy* OR horticultural* OR Indian* OR Kampo* OR Korean* OR Latin-American* OR laughter* OR Mongolian* OR Native-American* OR oriental* OR Tibetan* OR Arabic* OR Vietnamese* OR Asian* OR manipulat* OR holistic OR integrative*) NEAR/6 (medicine OR therap* OR practice* OR ritual* OR remed*)) OR cupping* OR dry-needling* OR enzyme-replacement* OR food-fad* OR forest-bathing* OR glucose-infusion* OR iridolog* OR iron-therap* OR mesotherap* OR moxibustion* OR naturopath* OR nutritional-support* OR orthomolecular* OR osteopath* OR oxygen-therap* OR prolotherap* OR psychodermatolog* OR reflexotherap* OR Shamanism* OR speleotherap* OR spiritual-healing* OR helminth* OR Aromatherap* OR Bach-flower OR breathwork OR herb* OR reiki OR fatty-Acid* OR omega-3* OR omega-6* OR pufa* OR ((digital* OR diet*) NEAR/3 (therap* OR intervention*)) OR ((plant) NEAR/3 (extract* OR medicinal*)) OR iron OR vitamin* OR multivitamin* OR magnesium* OR amino-acid* OR lipid* OR ((diet OR nutrition*) NEAR/3 (enriched* OR rich* OR addition* OR additive* OR mediteran*)) OR ((fish) NEAR/3 (consumption*)) OR fish-oil* OR olive-oil*):ab,ti)

AND

('systematic review'/de OR 'systematic review (topic)'/de OR 'meta analysis'/de OR 'meta analysis (topic)'/de OR ((systematic* NEAR/3 review*) OR meta-analy*):ab,ti) NOT [conference abstract]/lim

Non-invasive brain stimulation and bright light therapy interventions: Cochrane Database of Systematic Reviews search

Limits: 2010 onwards

(MeSH - Depression, Postpartum AND ((antenatal OR "ante natal" OR antepartum OR "ante partum" OR birth* OR breastfe* OR childbearing OR childbirth* OR labor OR labour OR lactat* OR matern* OR mother* OR parturition OR perinatal OR peripartum OR postnatal OR "post natal" OR postpartum OR "post partum" OR pregnan* OR prenatal OR "pre natal") AND (depress*)):ti,ab,kw)

AND

((((light) NEAR/2 (therap* OR treatment*) OR ultraso* OR biofeed* OR neurofeed*) OR ("NIBS" OR (("non invasive brain" OR "noninvasive brain" OR transcranial OR theta) NEAR/5 stimulat*) OR "tDCS" OR "tES" OR "ECT" OR "rTMS" OR "iTBS" OR "cTBS" OR "accelerated TMS" OR "electroconvulsive therap*")):ti,ab,kw)

PRISMA diagram. Treatment of PPD: Complementary and alternative treatments/Non-invasive brain stimulation interventions

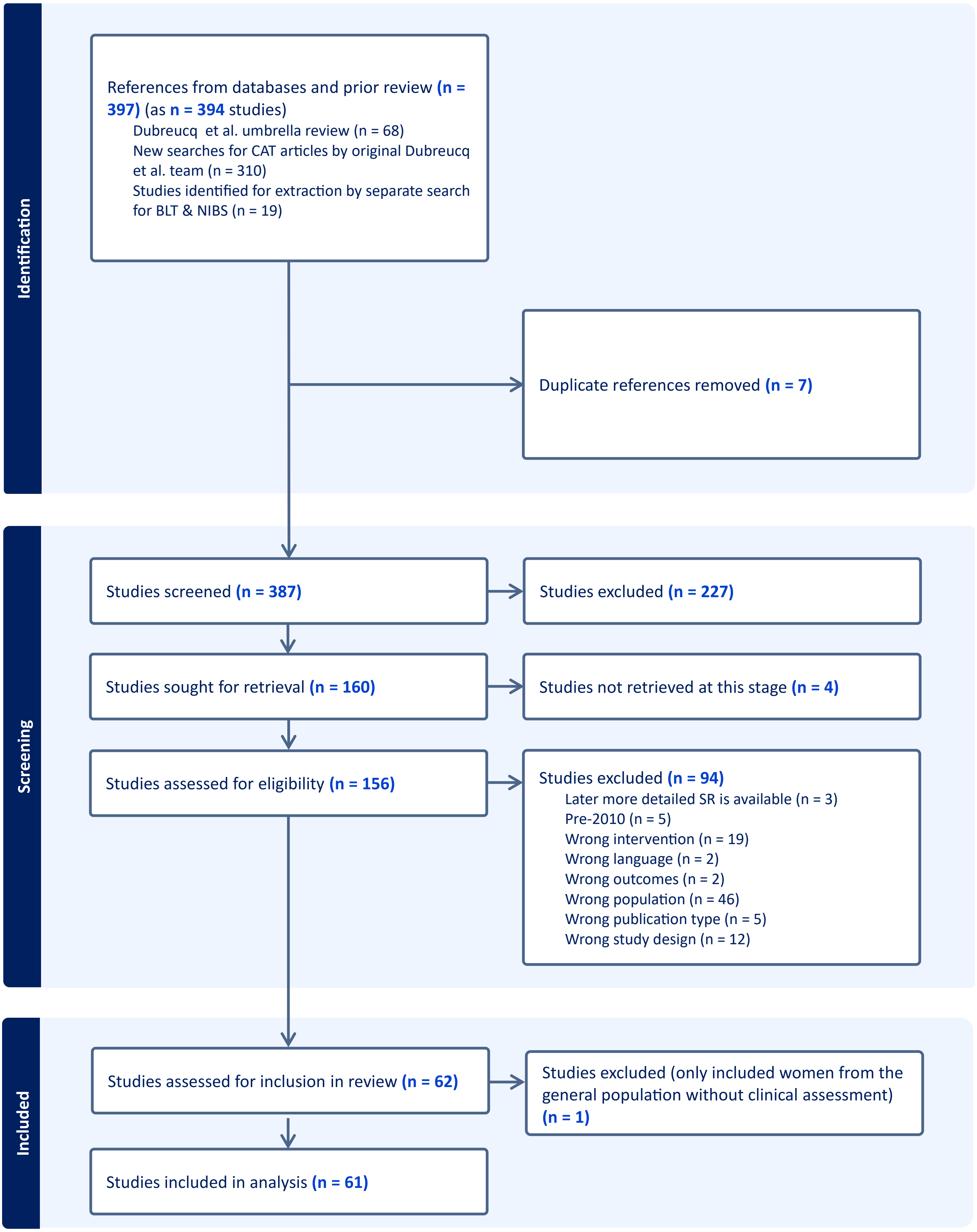

Supplement: Radoš et al. supplementary material 1 — Radoš et al. supplementary material [file S0007125025000431sup001.pdf]
